# Has COVID-19 Changed the Incidence and Profile of Late Presenters for HIV Infection in Lodz, Polish Reference Centre, Poland?

**DOI:** 10.3390/jcm13144121

**Published:** 2024-07-14

**Authors:** Kamila Wójcik-Cichy, Anna Piekarska, Elżbieta Jabłonowska

**Affiliations:** Department of Infectious Diseases and Hepatology, Medical University of Lodz, Kniaziewicza 1/5, 91-347 Łódź, Poland; annapiekar@op.pl (A.P.); elajablonowska@gmail.com (E.J.)

**Keywords:** COVID-19 pandemic, HIV late presenters, advanced late presenters, entry into HIV care, antiretroviral therapy

## Abstract

**Objectives**: The aim of this study was to compare the prevalence and characteristics of HIV late presenters (LPs) and advanced LPs (aLPs) registered in the Lodz HIV centre during the COVID-19 pandemic (2020–2021) with those of the pre-pandemic period (2017–2019). **Methods**: A retrospective analysis was performed of the predictive factors associated with HIV LPs and aLPs based on multivariable logistic regression. The patient entry into specialist HIV care after diagnosis during the pandemic was analysed. **Results**: Of 121 newly diagnosed HIV infections during the pandemic, 49.6% had late presentation and 36.4% had advanced HIV disease (AHD). In the pre-pandemic period, out of 154 newly diagnosed patients, 58.4% were LPs and 38.3% were aLPs. Independent risk factors for HIV late presentation were older age (OR: 1.04, 95% CI: 1.01–1.076; *p* = 0.008), diagnosis in hospital (OR: 5.63, 95% CI: 2.87–11.05; *p* < 0.001) and negative VDRL as compared to positive VDRL (OR: 2.48, 95% CI: 1.19–5.15; *p* = 0.015). The same predictive factors were associated with aLPs: older age (OR: 1.07, 95% Cl 1.04–1.11; *p* < 0.001), HIV diagnosis in hospital (OR: 4.25, 95% CI 2.17–8.29; *p* < 0.001) and negative VDRL as compared to positive VDRL (OR: 4.95, 95% CI 1.87–13.10; *p* = 0.001). HIV diagnosis during the pandemic was not a risk factor for late presentation nor for advanced late presentation. However, the time between HIV diagnosis and the first visit to an HIV centre was statistically lower in the pre-pandemic period (*p* = 0.0048); the median lengths of time between the date of HIV testing, the first visit to the centre and the initiation of ART did not differ between these two periods in LPs and aLPs (*p* > 0.05). **Conclusions**: The COVID-19 pandemic did not change the prevalence or characteristics of late presentation and aLPs among newly diagnosed patients, nor did it extend the time to enrolment in HIV care or ART introduction in these groups.

## 1. Introduction

The coronavirus (COVID-19) pandemic had a significantly negative impact on the healthcare system, particularly for patients with chronic conditions, including those with HIV infections. Up to 70% of HIV-outpatient clinics in Eastern Europe were disrupted, with 60% of HIV physicians directly involved in medical care for patients with COVID-19 [1]. Moreover, in many HIV centres across Europe, many routine clinical visits were replaced by telehealth visits [2]. A survey of over 98 outpatient HIV-Services in 53 European countries showed a reduction in HIV testing of up to 50% in 2020 [3].

Early HIV diagnosis, the initiation of antiretroviral therapy (ART) and long-term retention in care are known to decrease mortality rates in patients living with HIV [4]. Late HIV diagnosis correlates with a worse prognosis and is associated with both the occurrence of opportunistic infections and an increase in the prevalence of diseases not related to AIDS, such as cardiovascular diseases or cancers [5,6,7]. Results from PISCID and ICONA cohort studies show that the late diagnosis of HIV is associated with increased mortality and poorer response to ART treatment. Moreover, the favourable CD4 cell recovery from ART has a positive influence on survival among late presenters [8,9].

The European Late Presenter Consensus working group defines late presentation as a diagnosis of HIV with a CD4 count ≤ 350 cell/μL or the occurrence of an AIDS-defining event, regardless of the CD4 cell count. A diagnosis of advanced HIV disease (AHD) is also classified as a CD4 cell count below 200 cells/μL or with an AIDS-defining event at the first follow-up [10].

The European Centre for Disease Prevention and Control estimated the percentage of HIV late presenters (LPs) in Europe in 2010–2016 to range from 38.3% to 64.2% [11]. In a large multicentre Polish cohort consisting of 3972 patients newly diagnosed with HIV who were followed up on from 2000–2015, 57.6% of the patients entered care late, with a third presenting with AHD [12].

The predictive factors for late HIV presentation vary between study populations [12,13,14,15,16,17]. In Polish studies, older age, female sex, injecting drug use, a heterosexual route of HIV transmission, a positive anti-HCV result and latent syphilis infection were found to influence HIV late presentation [12,13,18].

The present study is the first in Poland to assess the prevalence of HIV late presenters (LPs) and risk factors for HIV late presentation during the COVID-19 pandemic. It is also the first study to examine the standard of HIV care during this pandemic in Poland.

## 2. Aims

The aim of this study was to compare the prevalence and characteristics of HIV late diagnosis and AHD during the COVID-19 pandemic (2020 and 2021) with the pre-COVID-19 period (2017–2019) among patients with newly-diagnosed HIV infections who were registered in a HIV centre in Lodz; it also identifies the predictive factors associated with late diagnosis. The study also examines entry into specialist HIV care after diagnosis, measured as the time between the diagnosis of HIV, the first visit to the clinic and the start of antiretroviral treatment.

## 3. Materials and Methods

The participant group comprised patients with newly diagnosed HIV. All were registered in the AIDS centre in Lodz before the COVID-19 pandemic, i.e., from 2017 to 2019, and then during its course (2020–2021). The patients were grouped according to the period in which they were diagnosed with HIV. Of note, we also analysed a subgroup of new HIV-diagnosed Ukraine migrants who were registered in our centre during these years.

The records of all patients with a first diagnosis of HIV were analysed for cases of late presentation and AHD. The age, sex, route of transmission, AIDS category at diagnosis according to the 1993 Centres for Disease Control and Prevention case definition, migration data, HCV antibody level, HBs antigen, VDRL (Venereal Diseases Research Laboratory), HIV viral load and baseline CD4 count were collected at the time of diagnosis. The time to the first visit in the clinic after receiving a positive HIV immunoblotting or HIV viraemia result and to the introduction of ART were also recorded.

The inclusion criteria were as follows: new HIV diagnosis confirmed by positive immunoblotting or by serum HIV-RNA in patients with acute retroviral syndrome between 2017 and 2021, who have had at least one visit in a Lodz centre. The exclusion criteria were as follows: age under 18 years old and first follow-up visit after HIV diagnosis in another HIV centre. Prisoners were also excluded. This study was conducted in accordance with the Helsinki Declaration of 1975. The study protocol was approved by the Bioethical Committee of Medical University in Szczecin, Poland (approval number KB-006/40/23).

The following nominal variables were compared between the pre-pandemic and pandemic groups using the Chi-square test: gender, stage of HIV infection at the diagnosis, route of HIV transmission, HCV antibodies, HBsAg, VDRL, diagnosis in hospital and diagnosis during the COVID-19 period. The following continuous variables were analysed using the non-parametric Mann–Whitney *U*-test: age at HIV diagnosis, CD4 lymphocyte count, HIV viral load at care entry, time to care entry after receiving a positive HIV test and time to the introduction of ART. The factors associated with late presentation to care and the AHD for each group were analysed using multivariable logistic regression. The significance threshold was assumed as *p* < 0.05.

## 4. Results

### 4.1. Study Group Characteristics

In total, 275 persons aged 19–70 years (232 men and 43 women) were newly diagnosed as HIV-positive in the whole study period from 2017–2021. Acute retroviral syndrome was reported in six patients before the pandemic and only in one patient during the pandemic (*p* > 0.05). AIDS-defining diseases were diagnosed in 23 subjects (14.9%) in the pre-COVID-19 period and 25 (20.7%) during COVID-19. The demographics and clinical characteristics of the entire study group during the pre-COVID-19 and COVID-19 periods are presented in Table 1.

**Figure 1:**
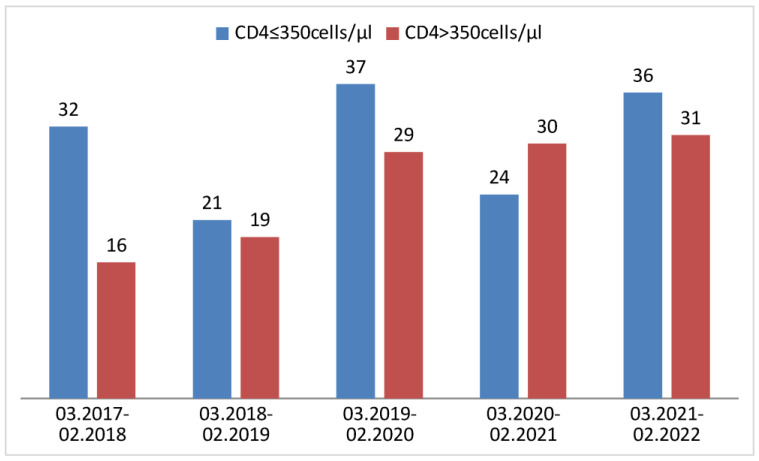
Proportion of LPs in particular years.

**Figure 2:**
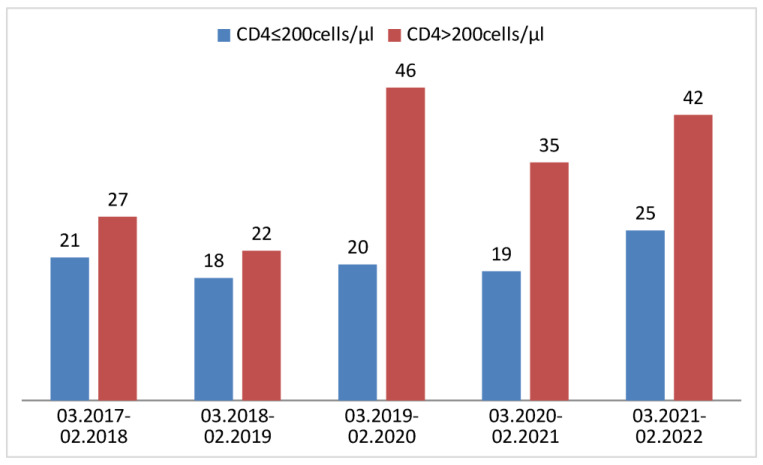
Proportion of AHD in particular years.

Of 121 newly diagnosed HIV infections in the pandemic, 49.6% had late presentation and 36.4% had advanced HIV disease. In the pre-pandemic period, out of 154 newly diagnosed patients, 58.4% were LPs and 38.3% were advanced LPs (aLPs). Figure 1 and Figure 2 show the proportion of LPs and aLPs in particular years.

No differences in the prevalence of late presentation and AHD were found between the pre-COVID-19 and COVID-19 periods. Again, the differences in age, sex, route of HIV transmission, presence of HCV antibodies and HBsAg between these two groups were statistically not significant.

### 4.2. Characteristics of Late Presenters and Factors Associated with Late Presentation

The late presenters (LPs) comprised 31 women (20.7%) and 119 men (97.3%) (Table 2). The univariable and multivariable logistic regression of the factors associated with advanced late presentation are shown in Table 3. Multivariable logistic regression found the independent risk factors for LPs to be older age (OR: 1.04, 95% CI: 1.01–1.076; *p* = 0.008), negative VDRL (compared to positive VDRL: OR: 2.48, 95% CI: 1.19–5.15; *p* = 0.015) and diagnosis in hospital (OR: 5.63, 95% CI: 2.87–11.05; *p* < 0.001). HIV diagnosis during the pandemic was not a risk factor for late presentation.

### 4.3. Characteristics of Advanced Late Presenters and Factors Associated with Advanced Late Presentation

The advanced late presenters comprised 103 people (37.45% of the study group): 24 women and 79 men (Table 4). The univariable and multivariable logistic regression of factors associated with advanced late presentation are shown in Table 5. The independent risk factors for advanced late presentation were older age (OR: 1.07, 95% Cl 1.04–1.11; *p* < 0.001), negative VDRL (compared to positive VDRL: OR: 4.95, 95% CI 1.87–13.10; *p* = 0.001) and HIV diagnosis in hospital (OR: 4.25, 95% CI 2.17–8.29; *p* < 0.001). HIV diagnosis during the pandemic was not associated with the risk of advanced HIV disease in newly diagnosed patients.

### 4.4. Ukrainian Patients

Due to the fact that these data were collected before the war in Ukraine in 2022, in total, only 35 Ukrainian patients were included in the analyses: 19 women (54.3%) and 16 men (45.7%). This group included a higher proportion of female patients than the non-Ukrainian group (*p* < 0.001). Within this group, heterosexual transmission (51.4%) was the predominant HIV transmission route and was reported more frequently than in Polish patients (*p* < 0.001). The newly diagnosed Ukraine patients were significantly older (*p* < 0.05) than the Polish patients, with a median age of 36.1 years (IQR 8.0) compared to 33 years (IQR 15.1). They were also more likely to obtain positive HCV antibody results compared to Polish patients (25.77% vs. 8.3%; *p* < 0.01).

In addition, 22 individuals were LPs (62.9%) and 16 subjects demonstrated AHD (45.7%). The median baseline CD4 count in the group (249 cells/μL; IQR 390) was lower than in the Polish patients (334.5 cells/μL; IQR 361) with borderline statistical significance (*p* = 0.058). However, AIDS-defining diseases were reported in 11 subjects (31.43%) and were significantly more frequent than in the Ukrainian subjects (*p* = 0.04).

A comparison of the Ukrainian and Polish subjects who entered care in the Lodz centre is presented in Table 6.

### 4.5. The Time to Enrolment in HIV Care and ART Introduction

The median time from HIV diagnosis to first visit in the whole group was 3 days during the pre-COVID-19 period (IQR 14) and 12 days during the pandemic (IQR 23). In our study group, the time between HIV diagnosis and first visit was statistically lower in the pre-pandemic period (*p* = 0.0048).

However, the impact of the COVID-19 pandemic on the time to first visit after HIV diagnosis was not found in the LPs and aLPs groups. In the LPs, the median lengths of time between the date of HIV testing and the first visit were 7 days (IQR 19) in the pre-pandemic period and 1 day (IQR 19) during the pandemic. In the aLPs, the median periods between the date of HIV testing and care entry were 1 day (IQR 13) in the pre-pandemic period and 4 days (IQR 18) during the pandemic. These differences were not statistically significant (Table 7).

The time of ARV initiation remained unaffected during the lockdown in the whole study group and in the LPs and aLPs. The median periods for the whole group between HIV diagnosis and antiretroviral initiation were 7 days (IQR 13) before COVID-19 and 7 days (IQR 2) during the pandemic. Again, this difference was not statistically significant.

The median periods between HIV diagnosis and antiretroviral initiation in the LPs and aLPs did not differ before COVID-19 or during the pandemic (Table 7).

## 5. Discussion

Late presentation continues to be an important issue in newly HIV-diagnosed subjects in the Lodz region.

In the period before the COVID-19 pandemic, i.e., from 2017 to 2019, 58.4% patients were LPs and 38.3% were advanced LPs.

However, the trend in HIV late presentation was relatively stable and did not change during the COVID-19 period. Of note, these values were lower than those noted previously in the Lodz centre from 2009–2016, where 62.9% patients were HIV LPs, and 43.2% were advanced LPs [18]. Similarly, lower percentages of LPs (44.8%) and advanced LPs (25.4%) were observed in the Test and Keep in Care (TAK) Project Poland between 2016 and 2017 [13]. However, our outpatient HIV clinic is so far the only one in the Lodz region; the main limitations of our study are the fact that this study included only the local population and that our results may not be representative for the whole Polish HIV-infected population.

Moreover, the literature data assessing the incidence of late presenters during the pandemic are sparse. A study of an Italian cohort and the AHF Global Quality Program revealed a significant reduction in HIV diagnoses, including late-stage presentations, during the pandemic [19,20]. However, such decreases may be associated with lockdown periods, restrictions and closures of voluntary counselling and testing services.

Due to the war in Ukraine, the number of HIV-positive migrants registered in our centre has grown in recent years. This trend was also observed before the Russia–Ukraine war. In the present study, 22 of the tested Ukrainian patients were LPs (62.9%), and AIDS was diagnosed in 11 subjects (31.43%). This notably high percentage of LPs and AIDS diagnoses in Ukrainian patients demonstrate that late presentation remains a challenge among migrants. Further efforts, such as routine HIV screening, must be made in such groups to ensure early diagnoses.

This study also assessed the risk factors for late-stage HIV diagnosis. Similarly to Xi Hu et al., our data indicates that LPs were more likely to be diagnosed in hospitals [21]. It is worth noting that seven patients in our group with suspected COVID-19 were diagnosed with AIDS.

Acute retroviral syndrome should also be considered in differential diagnoses for evaluating patients with acute viral syndromes, including COVID-19. However, only one patient with an early HIV infection was diagnosed during the COVID-19 pandemic. It is possible that symptomatic HIV conditions such as acute HIV infection or even AIDS-defining diseases were under-diagnosed during the pandemic. This may be associated with a lack of knowledge regarding recommended HIV testing among the patients with suspected SARS-CoV-2 infections, especially by non-HIV-specialist physicians. We speculate that the observed reduction in acute retroviral syndrome cases during the COVID-19 pandemic may be associated with lockdowns and the fear of SARS-CoV-2 infection. Overall, our findings are consistent with those from Italy and Spain, which also show a decline in the prevalence of other sexually transmitted infections (STIs), mainly syphilis, during the lockdown period [22,23].

Moreover, the use of telehealth visits in general practice might have led to misdiagnoses, even in symptomatic HIV patients. In addition, the closure of voluntary counselling and testing services from March to May 2020 in Poland and the disruption in their activities throughout 2020 were also associated with a decrease in the number of performed HIV tests during the pandemic [24]. Similarly to other authors’ findings, older age and negative VDRL status were found to be independent factors associated with the late presentation of HIV [12,13]. The lack of declaration of the route of HIV transmission was also associated with LP and AHD. We speculate that undisclosed transmission route reflects stigma and poses a barrier to HIV testing.

According to Spornraft–Ragaller et al., MSMs with positive syphilis tests showed a lower risk of late presentation [25]. It is not surprising that MSMs, with a higher perception of the risks of HIV infection, are less likely to be LPs [26]. Regular screenings for HIV and other STIs are recommended in the MSM group.

The pandemic had negative effects on health services across the globe. The reduction in the provision of medical care during the COVID-19 period resulted in delays in the diagnosis and management of all chronic diseases.

Little is known of the linkage to care among newly diagnosed HIV patients during the COVID-19 pandemic. Data from the Test and Keep in Care (TAK) Project Poland, which assessed care in the pre-pandemic period, show that 62.1% of people are linked to care after receiving a HIV diagnosis, and the majority started ART [27].

In the whole study group, a significant difference in the median length of time between the date of HIV testing and the first visit was found between the pre-COVID-19 period and the period during the COVID-19 pandemic. The lockdown restrictions and stay-at-home orders would have negatively affected access to medical care.

However, the impact of the COVID-19 pandemic on the time to first visit after HIV diagnosis was not reported in the LPs and aLPs groups. The symptomatic LPs are mainly diagnosed in hospital and require the rapid initiation of ART. Our study does not show the deleterious impact of the COVID-19 pandemic on the implementation of ART.

The time of ART initiation did not increase during the lockdown, suggesting that the functionality of our HIV outpatient clinic was generally maintained at the same level as during the pre-pandemic period.

Similar results were obtained by El Moussaoui et al., who did not observe any delay between HIV diagnosis and treatment initiation in 2020 as compared to 2019 [2].

## 6. Conclusions

The COVID-19 pandemic did not change the prevalence of LPs and aLPs or their characteristics. Moreover, the time to enrolment in HIV care and the introduction of ART in late presenters were not extended during the pandemic. Late presentation and advanced HIV disease remain common among newly diagnosed HIV patients, particularly migrants from Ukraine; massive public health strategies are needed for addressing emerging unknown HIV infections.

## Figures and Tables

**Table 1:** Patient characteristics before and during the COVID-19 period.

	Pre-COVID-19 N = 154		COVID-19 Period N = 121		
	Median/N	IQR/%	Median/N	IQR/%	Value
Male sex	130	84.4%	102	84.3%	>0.05
Age in years	33.6	15.2	33.6	9.7	>0.05
CD4 cells/μL	282.0	342.0	362.5	403.0	>0.05
Diagnosis in hospital	59	33.3%	33	27.3%	>0.05
Diagnosis in outpatient clinics, laboratories orVoluntary Testing and Counselling sites	95	66.7%	88	72.7%	>0.05
Diagnosis during pregnancy	5	3.2%	7	5.8%	>0.05
AIDS	23	14.9%	25	20.7%	>0.05
Advance late presenters	59	38.3%	44	36.4%	>0.05
Late presenters	90	58.4%	60	49.6%	>0.05
Acute retroviral syndrome	6	0.4%	1	0.8%	>0.05
**Transmission route**					>0.05
MSM	105	68.2%	80	66.1%	
HTX	32	20.8%	27	22.3%	
IDU	7	4.5%	4	3.3%	
undisclosed transmission route	10	6.5%	10	8.3%	
**HBV**					>0.05
positive	6	3.9%	4	3.30%	
negative	145	94.2%	116	95.9%	
unknown	3	1.9%	1	0.8%	
**HCV**					>0.05
positive	21	13.6%	8	6.7%	
negative	130	84.4%	112	92.6%	
unknown	3	1.9%	1	0.8%	
**VDRL**					>0.05
positive	24	15.6%	33	27.3%	
negative	124	80.5%	87	71.8%	
unknown	6	3.9%	1	1.0%	

**Table 2:** Characteristics of late presenters and persons who entered HIV care earlier.

	Late presenters N = 150		Non-Late Presenters N = 125		
	Median/N	IQR/%	Median/N	IQR/%	Value
Male sex	119	79.3%	113	90.4%	>0.05
Age in years	35.6	13.7	31.82	11.3	<0.01
Diagnosis in hospital	76	50.7%	16	12.8%	<0.001
Diagnosis in outpatient clinics, laboratories or Voluntary Testing and Counselling sites	74	49.3%	109	87.2%	
**Transmission route**					<0.001
MSM	86	57.3%	99	79.2%	
HTX	36	24.0%	23	18.4%	
IDU	11	7.3%	0	0%	
undisclosed transmission route	17	11.3%	3	2.4%	
**HBV**					>0.05
positive	8	5.3%	2	1.6%	
negative	140	93.3%	121	96.8%	
unknown	2	1.3%	2	1.6%	
**HCV**					>0.05
positive	18	12.0%	11	8.8%	
negative	130	86.7%	112	89.6%	
unknown	2	1.3%	2	1.6%	
**VDRL**					<0.001
positive	18	12.0%	39	31.2%	
negative	127	84.7%	84	67.2%	
unknown	5	3.3%	2	1.6%	

**Table 3:** Univariable and multivariable logistic regression of the factors associated with late presentation.

	Late Presenters N = 150			Non-Late Presenters N = 125		
	Multivariable *			Univariable		
	OR	95% CI	Value	OR	95% CI	Value
Male sex	0.83	0.28–2.48	0.735	0.41	0.20–0.84	0.015
Age in years	1.04	1.01–1.076	0.008	1.06	1.03–1.09	<0.001
Diagnosis in hospital	5.63	2.87–11.05	<0.001	7.04	0.99–49.94	0.051
COVID-19 period	0.87	0.49–1.54	0.641	0.69	0.43–1.13	0.140
Ukrainian nationality	1.09	0.41–2.86	0.863	1.39	0.20–9.86	0.742
**Transmission route**						
MSM	1			1		
HTX	1.36	0.55–3.34	0.503	1.85	1.01–3.38	0.047
IDU	8.03	0.80–80.33	0.0762	12.5	1.57–99.71	0.017
undisclosed transmission route	2.28	0.51–10.10	0.279	7.92	2.26–27.70	0.001
**HBV**						
positive	1			1		
negative	0.52	0.08–3.37	0.497	0.31	0.06–1.53	0.150
unknown	0.28	0.00–41.43	0.621	0.29	0.02–3.52	0.328
**HCV**						
positive	1			1		
negative	2.00	0.64–6.23	0.233	0.70	0.32–1.57	0.390
unknown	1.14	0.009–132.56	0.958	0.65	0.08–5.29	0.685
**VDRL**						
positive	1			1		
negative	2.48	1.19–5.15	0.015	2.99	1.60–5.57	<0.001
unknown	8.26	0.70–96.70	0.093	10.83	1.18–99.59	0.035

* Model adjusted for all above.

**Table 4:** Characteristics of advanced late presenters and persons who entered HIV care earlier.

	Advanced Late Presenters N = 103		Non-Advanced Late Presenters N = 172		
	Median/N	IQR/%	Median/N	IQR/%	Value
Male sex	79	76.7%	153	89.0%	<0.01
Age in years	38.9	13.6	30.98	11.1	<0.001
Diagnosis in hospital	58	56.3%	34	19.8%	<0.001
Diagnosis in outpatient clinics, laboratories or Voluntary Testing and Counselling sites	45	43.7%	138	80.2%	
**Transmission route**					<0.001
MSM	51	49.5%	134	77.9%	
HTX	27	26.2%	32	18.6%	
IDU	8	7.8%	3	1.7%	
undisclosed transmission route	17	16.5%	3	1.7%	
**HBV**					>0.05
positive	7	6.8%	3	1.7%	
negative	94	91.3%	167	97.0%	
unknown	2	1.9%	2	1.2%	
**HCV**					<0.05
positive	17	16.5%	12	7.0%	
negative	84	81.6%	158	91.9%	
unknown	2	1.9%	2	1.2%	
**VDRL**					<0.001
positive	7	6.8%	50	29.1%	
negative	93	90.3%	118	68.6%	
unknown	3	2.9%	4	2.3%	

**Table 5:** Univariable and multivariable logistic regression of the factors associated with advanced late presentation.

	Advanced Late Presenters N = 103			Non-Advanced Late Presenters N = 172		
	Multivariable *			Univariable		
	OR	95% CI	Value	OR	95% CI	Value
Male sex	0.70	0.24–2.09	0.527	0.44	0.22–0.85	0.016
Age years	1.07	1.04–1.11	<0.001	1.09	1.06–1.13	<0.001
Diagnosis in hospital	4.25	2.17–8.29	<0.001	5.23	3.02–9.05	<0.001
COVID-19 period	1.27	0.67–2.41	0.463	0.90	0.55–1.48	0.678
Ukrainian nationality	0.61	0.21–1.78	0.369	1.54	0.22–10.95	0.665
**Transmission route**						
MSM	1			1		
HTX	1.30	0.52–3.25	0.581	2.21	1.19–4.11	0.012
IDU	2.55	0.46–14.06	0.282	7.55	1.92–29.64	0.004
undisclosed transmission route	5.35	1.15–24.83	0.032	17.92	5.07–63.33	<0.001
**HBV**						
positive	1			1		
negative	0.46	0.08–2.70	0.388	0.27	0.07–1.12	0.072
unknown	0.52	0.00–205.66	0.828	0.50	0.05–5.51	0.571
**HCV**						
positive	1			1		
negative	0.69	0.22–2.14	0.517	0.39	0.17–0.86	0.019
unknown	0.84	0.00–285.19	0.954	0.75	0.09–6.11	0.788
**VDRL**						
positive	1			1		
negative	4.95	1.87–13.10	0.001	5.39	2.33–12.45	<0.001
unknown	2.57	0.20–33.28	0.469	7.14	1.20–42.57	0.031

* Model adjusted for all above.

**Table 6:** Comparison of the Ukrainian and non-Ukrainian patients.

	Ukrainian Nationality N = 35		Not Ukrainian Nationality N = 240		
	Median/N	IQR/%	Median/N	IQR/%	p Value
Male sex	16	45.7%	216	90.0%	<0.001
Age in years	36.1	8.0	33.0	15.1	<0.05
CD4 cells/μL	249	390.0	334.5	361.0	=0.058
Diagnosis in hospital	14	40.0%	78	32.5%	>0.05
Diagnosis in outpatient clinics, laboratories or Voluntary Testing and Counselling sites	21	60.0%	162	67.5%	>0.05
Diagnosis in COVID-19 period	18	51.4%	103	42.9%	>0.05
Diagnosis in pre-COVID-19 period	17	48.6%	137	57.1%	>0.05
AIDS	11	31.43%	37	15.41%	=0.04
Advance late presenters	16	45.7%	87	36.3%	>0.05
Late presenters	22	62.9%	128	53.3%	>0.05
**Transmission route**					<0.001
MSM	10	28.6%	175	72.9%	
HTX	18	51.4%	41	17.1%	
IDU	2	5.7%	9	3.8%	
undisclosed transmission routes	5	14.3%	15	6.3%	
**HBV**					>0.05
positive	2	5.7%	8	3.3%	
negative	33	94.3%	228	95.0%	
unknown	0	0%	4	1.7%	
**HCV**					<0.01
positive	9	25.7%	20	8.3%	
negative	26	74.3%	216	90.0%	
unknown	0	0%	4	1.67%	
**VDRL**					>0.05
positive	4	11.4%	53	22.1%	
negative	31	88.6%	180	75%	
unknown	0	0%	7	2.9%	

**Table 7:** Linkage to care in advanced late presenters and late presenters.

	Pre-COVID-19 Period N = 154	COVID-19 Period N = 121	*p*
**Study group**			
Days between date of testing and first visit	3 (14)	12 (23)	=0.0048
Days between first visit and treatment initiation	7 (13)	7 (2)	>0.05
**Advanced late presenters**	**Median (IQR)**	**Median (IQR)**	*p*
Days between date of testing and first visit	1 (13)	4 (18)	>0.05
Days between first visit and treatment initiation	7 (11.5)	3 (6)	>0.05
**Late presenters**			
Days between date of testing and first visit	7 (19)	1 (19)	>0.05
Days between first visit and treatment initiation	7 (13)	4.5 (9)	>0.05

## Data Availability

Data Availability Statements are available in section “MDPI Research Data Policies” at https://www.mdpi.com/ethics (accessed on 10 July 2024).
